# A dual role for hypoxia inducible factor-1α in the hepatitis C virus lifecycle and hepatoma migration

**DOI:** 10.1016/j.jhep.2011.11.018

**Published:** 2012-04

**Authors:** Garrick K. Wilson, Claire L. Brimacombe, Ian A. Rowe, Gary M. Reynolds, Nicola F. Fletcher, Zania Stamataki, Ricky H. Bhogal, Maria L. Simões, Margaret Ashcroft, Simon C. Afford, Ragai R. Mitry, Anil Dhawan, Christopher J. Mee, Stefan G. Hübscher, Peter Balfe, Jane A. McKeating

**Affiliations:** 1 Institute for Biomedical Research, College of Medical and Dental Sciences, University of Birmingham, Birmingham, United Kingdom; 2 Division of Medicine, University College London, London, United Kingdom; 3 Institute of Liver Studies, Kings College Hospital and Kings College London School of Medicine, London, United Kingdom; 4 Department of Cellular Pathology, Queen Elizabeth Hospital Birmingham, Birmingham, United Kingdom

**Keywords:** BC, bile canaliculi, CMFDA, 5-chloromethylfluorescein diacetate, HCC, hepatocellular carcinoma, EMT, epithelial to mesenchymal transition, HCVcc, hepatitis C virus cell culture, HIF-1α, hypoxia inducible factor 1 alpha, JFH-1, Japanese fulminant hepatitis-1, MRP-2, multidrug resistant protein-2, PHH, primary human hepatocytes, SR-BI, scavenger receptor class B member 1, TGFβ, transforming growth factor-beta, TNFα, tumor necrosis factor alpha, VEGF, vascular endothelial growth factor, VSV-G, vesicular stomatitis virus glycoprotein, Hepatitis C, Hypoxia, Invasion

## Abstract

**Background & Aims:**

Hepatitis C virus (HCV) causes progressive liver disease and is a major risk factor for the development of hepatocellular carcinoma (HCC). However, the role of infection in HCC pathogenesis is poorly understood. We investigated the effect(s) of HCV infection and viral glycoprotein expression on hepatoma biology to gain insights into the development of HCV associated HCC.

**Methods:**

We assessed the effect(s) of HCV and viral glycoprotein expression on hepatoma polarity, migration and invasion.

**Results:**

HCV glycoproteins perturb tight and adherens junction protein expression, and increase hepatoma migration and expression of epithelial to mesenchymal transition markers Snail and Twist via stabilizing hypoxia inducible factor-1α (HIF-1α). HIF-1α regulates many genes involved in tumor growth and metastasis, including vascular endothelial growth factor (*VEGF*) and transforming growth factor-beta (*TGF-β*). Neutralization of both growth factors shows different roles for VEGF and TGFβ in regulating hepatoma polarity and migration, respectively. Importantly, we confirmed these observations in virus infected hepatoma and primary human hepatocytes. Inhibition of HIF-1α reversed the effect(s) of infection and glycoprotein expression on hepatoma permeability and migration and significantly reduced HCV replication, demonstrating a dual role for HIF-1α in the cellular processes that are deregulated in many human cancers and in the viral life cycle.

**Conclusions:**

These data provide new insights into the cancer-promoting effects of HCV infection on HCC migration and offer new approaches for treatment.

## Introduction

Hepatocellular carcinoma (HCC) is the most common liver malignancy and rates fifth in incidence and third in mortality in the world [1,2]. HCC is a complex and heterogeneous tumor with frequent intrahepatic spread and extrahepatic metastasis, resulting in poor prognosis [3]. Our current understanding of the molecular mechanisms underlying HCC pathogenesis is limited and further studies are required to aid the design of strategies for HCC treatment.

Hepatitis C virus (HCV) induces chronic liver injury that can lead to progressive fibrosis and is one of the leading causes of HCC [4]. HCV is a positive stranded RNA flavivirus that infects hepatocytes and replicates in the cytoplasm without integration into the host genome. The role of HCV infection in the carcinogenic process is unclear, in part due to the limited availability of small animal models that support HCV replication and technical difficulties in detecting HCV infected cells in the human liver [5]. Reports, demonstrating that HCV encoded proteins interact with cell cycle regulators and tumor suppressors, along with the development of HCC in some HCV transgenic lineages, suggest that HCV proteins may be directly oncogenic (reviewed in [6]). HCV-associated HCC has been reported to be associated with an increased recurrence after liver resection [7], suggesting that HCV may promote tumor growth and metastasis.

Recent advances allowing the assembly of infectious HCV particles *in vitro* have enabled the complete virus lifecycle to be studied [8]. HCV encodes two glycoproteins E1 and E2 that mediate virus attachment to the host cell receptors: tetraspanin CD81 and scavenger receptor class BI (SR-BI). More recently, tight junction proteins Claudin-1 and Occludin have been implicated in HCV entry (reviewed in [9]). We previously reported that HCV infection reduced hepatoma polarity reminiscent of epithelial to mesenchymal transition (EMT) [10]. Given the many reports detailing aberrant tight junction protein expression and EMT in malignant neoplasms including HCC [11–13] and the knowledge that viruses frequently down regulate expression of their cellular receptors, we investigated the effect(s) of HCV infection on hepatoma migration and invasion. We demonstrate that HCV glycoproteins and virus infection reduce tight junction integrity and E-Cadherin expression, promote EMT markers Snail and Twist expression and hepatoma migration via stabilizing hypoxia inducible factor 1a (HIF-1α), a transcriptional regulator that activates vascular endothelial growth factor (VEGF) and transforming growth factor (TGFβ) expression. We demonstrate a role for VEGF and TGFβ in de-regulating hepatoma polarity and promoting the migration of infected cells. Inhibition of HIF-1α reversed the effect(s) of virus glycoproteins and infection on hepatoma migration and significantly reduced HCV replication, demonstrating a dual role for HIF-1α in deregulating cellular processes associated with tumor growth and in the viral life cycle. HIF-1α is expressed in many human cancers and is considered a therapeutic target for treating malignant diseases of diverse aetiologies. These data provide new insights into the role for HCV in HCC pathogenesis and suggest new approaches for treatment.

## Materials and methods

### Cell lines, antibodies and cytokine assays

HepG2 and Huh-7.5 were propagated in Dulbecco’s Modified Eagle’s medium supplemented with 10% fetal bovine serum (FBS) and 1% non-essential amino acids. HepG2 cells were transduced to express human CD81, as previously reported [14]. Human hepatocytes were isolated according to previously published protocols [15] and maintained in Williams E medium supplemented with 10% FBS/5 mM HEPES/insulin/dexamethasone.

The following antibodies were used: anti-multi-drug resistant protein-2 (MRP-2), anti-vesicular stomatitis virus glycoprotein (VSV G) (Abcam), anti-NS5A 9E10, anti-CD81 2s131, anti-SR-BI (BD Biosciences), anti-Occludin and Claudin-1 (Invitrogen), anti-HIF-1α (Novus Biologicals), anti-VEGF VG76e, and anti-HCV E2 3/11 [16]. Alexa-conjugated secondary antibodies were purchased from Invitrogen. HIF-1α inhibitors NSC-134574 (NSC) and RITA were previously reported [17,18]. Human VEGF and TGFβ were measured by ELISA (Peprotech, UK) according to the manufacturer’s instructions.

### Expression of HCV and VSV glycoproteins

HepG2-CD81 cells were transfected with 4 μg of pcDNA3.1 plasmid encoding HCV strain H77 E1E2 (genotype 1), JFH-1 E1E2 (genotype 2) or VSV-G using magnet assisted transfection (Fisher Scientific, UK). Plasmids encode H77 or JFH-1 amino acids 171–747, spanning the leader sequence of E1 and the entire E1E2 coding region (VBRC HCV database numbering system). Seventy-two hours post transfection, the medium was replaced with 3% FBS/G418 (1 mg/ml) and glycoprotein expression confirmed by flow cytometry.

### Quantification of hepatoma polarity and tight junction integrity

Parental, HCV glycoprotein and VSV-G-expressing cells were grown for 5 days in culture to polarize before fixation with paraformaldehyde. Cells were permeabilized with PBS/0.5% BSA/0.1% Triton and stained for the apical marker MRP-2. Nuclei were visualized with 4′,6′diamidino-2-phenylindole (DAPI) and polarity index determined as the number of MRP-2 positive bile canalicular (BC) structures per 100 nuclei in 5-fields of view [19]. To assess tight junction integrity, polarized cells were incubated with 5 mmol/L 5-chromomethylfluorescein diacetate (CMFDA) at 37 °C for 15 min to allow translocation to BC. Following extensive washing in PBS, the ability of BC to retain CMFDA was measured as an indicator of tight junction integrity.

### Confocal microscopy

Cells were grown on 13 mm borosilicate coverslips, methanol fixed and permeabilized for 20 min in PBS/0.5% BSA/0.05% saponin. Cells were incubated with primary antibodies specific for Claudin-1, Occludin, HIF-1α, Snail, Twist or NS5A (1 μg/ml) for 1 h. After a saponin/BSA/PBS wash, cells were incubated with Alexa-conjugated secondary antibodies for 30 min. Cells were counterstained with DAPI and mounted onto glass slides using ProLong Gold antifade (Invitrogen). Laser scanning confocal microscopy was performed using a Zeiss Metahead confocal microscope with a 63× water objective.

### Human tissue and immunohistochemistry

Formalin-fixed paraffin embedded specimens were obtained from patients with early and late stage liver disease, diagnosed according to the severity of fibrosis, where early = no/mild fibrosis (Ishak stage <2) and late = cirrhosis. Normal tissue was obtained from surplus donor tissue used for reduced-size liver transplantation. Informed consent was obtained together with regional ethics committee approval. Immunohistochemical techniques were previously reported [20]. Microscopic examinations were performed by two independent observers. Occludin distribution was quantified on a scale of 0–3 according to its location at apical or basolateral membranes. The distribution in 5-fields of view from five cases for each disease was scored, defined as the percentage of hepatocytes expressing Occludin at apical or basolateral membranes, where 0 = <5%, 1 = 5–33%, 2 = 33–66% and 3 = >66%.

### HCV infection

HCV J6/JFH-1 virus was generated as previously described [21]. Cells were infected for 72 h under hypoxic (1% O_2_) or normoxic (20% O_2_) conditions in the presence or absence of HIF-1α inhibitor NSC (1 μM), previously shown to have a minimal effect on cell proliferation. Infected cells were detected by methanol-fixation and staining for HCV NS5A with mAb 9E10 and Alexa-594 anti-mouse. HCV RNA was quantified by RT-PCR following manufacturer’s guidelines (Invitrogen) using an MxPro 3000 real time PCR machine (Stratagene). Housekeeping gene *GAPDH* was included as an internal endogenous control for amplification efficiency and RNA quantification.

### Cell migration and invasion

HepG2 migration was assessed in a wound healing assay, where a scratch wound was created in the cell monolayer and cells re-fed with media containing mitomycin C (10 μg/ml) in the presence or absence of NSC (1 μM), anti-TGFβ or anti-VEGF (1.5 μg/ml). Cell migration was measured by taking an image of the scratch from replicate wells at 0 and 24 h post wounding. The wound closure distance was analyzed with ILab 4.0 software and the difference between 0 and 24 h calculated.

To assess hepatoma invasion, Huh-7.5 cells were serum starved overnight and seeded on collagen coated 8 μm transwell permeable membranes (BD, Falcon). Cells were allowed to invade for 24 h in the presence or absence of NSC (1 μM) and non-invading cells removed using a cotton-bud. Invaded cells were fixed in ice-cold methanol for 5 min, stained with 0.1% crystal violet for 1 h followed by washing in PBS and three fields of view captured using a Nikon TE200 microscope and the number of invaded cells enumerated.

### Statistical analysis

Results are expressed as mean ± 1 standard deviation, unless otherwise stated. Statistical analyses were performed using the Student *t* test, (unless otherwise stated) with a *p* value of less than 0.05 considered significant.

## Results

### HCV glycoproteins modulate tight junction protein localization, expression and function

**Supplementary Fig. 1:**
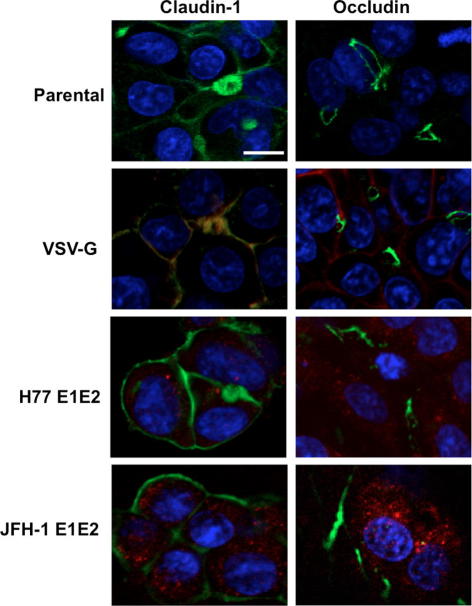
**HCV E2 and tight junction protein localization in HepG2 cells.** Staining of HCV E2 or VSV-G (red) and Claudin-1 or Occludin (green) in parental and HepG2 clones expressing VSV G, HCV strain H77 and JFH-1 glycoproteins. Cell nuclei were counterstained with DAPI (blue) and the scale bar represents 20 μm.

**Fig. 1:**
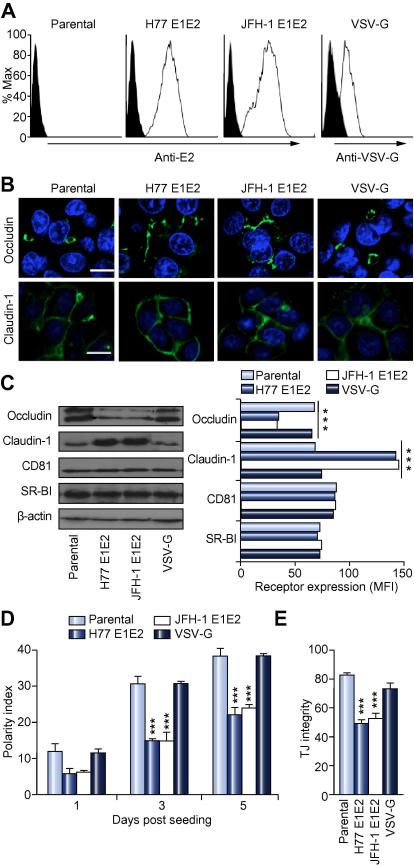
**HCV glycoproteins perturb tight junction function**. (A) Flow cytometric detection of HCV-E2 and VSV-G, where the filled histogram depicts an irrelevant isotype control. (B) Occludin and Claudin-1 (green) localization in parental, HCV glycoprotein and VSV-G expressing cells, where cell nuclei are counterstained with DAPI (blue); scale bar represents 20 μm. (C) HCV receptor quantification by Western blot and flow cytometry, where data is presented as mean fluorescent intensity (MFI) and isotype control value was subtracted. (D) HCV glycoproteins modulate polarity; cells were grown for 1, 3 and 5 days and their polarity assessed by enumerating MRP-2 positive BC/100 nuclei in five independent fields of view. (E) HCV glycoproteins reduce tight junction (TJ) integrity; cells were allowed to polarize for 5 days and tight junction integrity quantified by determining the number of BC retaining CMFDA. ^∗∗∗^*p* <0.001.

To investigate the functional effect(s) of HCV glycoproteins on tight junction proteins, we utilized the well-characterized human HepG2 hepatoblastoma cell line that polarizes in culture [19]. HepG2 cells were transfected to express HCV strains H77 and JFH-1 glycoproteins or control VSV-G (Fig. 1A). Stable cell populations were selected for study where the majority of cells expressed HCV E2 at comparable levels to HCVcc H77/JFH-1 infected hepatoma cells. Tight junction protein expression and localization in parental and transfected cells was assessed by confocal microscopy, Western blotting and flow cytometry (Fig. 1B and C). Occludin localized as discrete bands surrounding the BC in parental and VSV-G expressing cells, whereas in H77 and JFH-1 E1E2-expressing cells, Occludin localized to the apical and basolateral membranes and showed reduced expression levels by Western and flow cytometry (Fig. 1B and C). In contrast, HCV glycoproteins promoted Claudin-1 expression, with no effect on protein localization (Fig. 1 B and C). There was no discernable co-localization of HCV E2 with Claudin-1 or Occludin (Supplementary Fig. 1). HCV glycoproteins had no detectable effect on the expression level of entry factors CD81 and SR-BI (Fig. 1C). HCV glycoproteins significantly reduced HepG2 polarity (Fig. 1D) and the ability of BC to retain CMFDA (Fig. 1E), demonstrating an increased permeability. In summary, these data highlight a role for HCV glycoproteins in perturbing tight junction protein expression, localization and function that may result in an abnormal ‘leaky’ hepatic barrier *in vivo*.

**Fig. 2:**
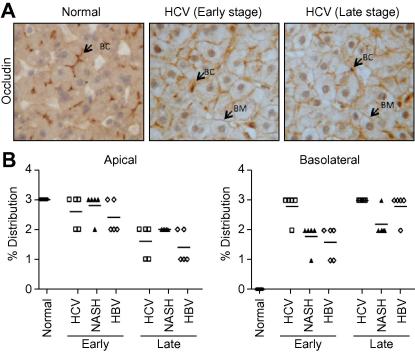
**Occludin localization in normal and diseased liver tissue**. (A) Representative immunohistochemical stain of Occludin in the normal, early and late stage HCV infected liver (200×), where the arrow shows Occludin at bile canalicular (BC) or basolateral (BM) hepatocyte membranes. (B) Occludin distribution in five cases of normal, HCV infected, NASH inflamed or HBV infected tissue was graded as follows: 0 = <5%; 1 = 5–33%; 2 = 33–66% and 3 = >66%. The basolateral distribution in all inflamed tissue was significantly higher compared to normal tissue.

To investigate whether HCV infection perturbs Occludin localization *in vivo*, formalin-fixed paraffin-embedded specimens of human liver samples from normal, HCV infected, non-alcoholic steatohepatitis (NASH) and hepatitis B virus (HBV) infected donors with early and late stage liver disease were stained for Occludin. In normal tissue, Occludin was only detected at the apical canalicular membrane, whereas basolateral pools were detected in all inflamed liver tissue samples, consistent with a reduction in apical expression (Fig. 2). The widespread reorganization of hepatocellular Occludin observed in all samples, independent of disease aetiology, suggests an indirect inflammatory response that is not specific to the HCV infected liver.

### Mechanism(s) underlying HCV glycoprotein modulation of tight junction function

**Supplementary Fig. 2:**
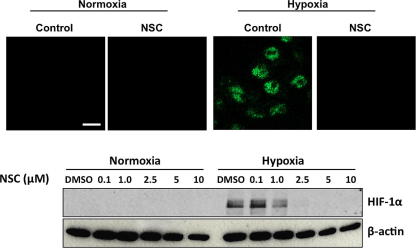
**NSC inhibition of HIF-1α.** Confocal microscopic imaging and western blot detection of HIF-1α in control or NSC (1 μM) treated HepG2-CD81 cells grown under hypoxic or normoxic conditions for 16 h. The scale bar represents 20 μm.

**Fig. 3:**
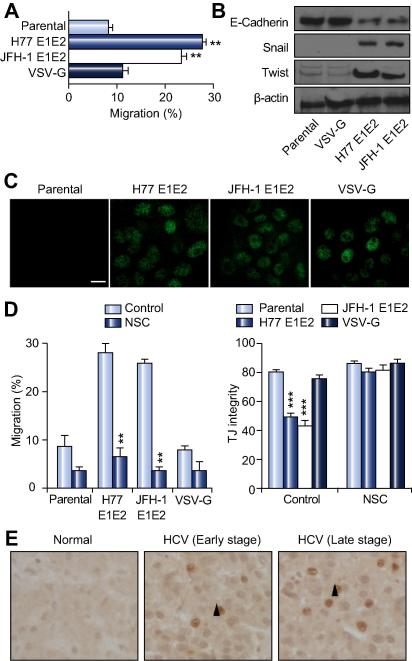
**HCV glycoproteins promote hepatoma migration**. (A) Increased migration of HCV glycoprotein expressing HepG2 cells; where migration was determined over a 24 h period in a scratch-wound assay. Data is presented as percentage migration. (B) Expression of E-Cadherin and EMT markers Snail and Twist. (C) HCV glycoproteins and VSV-G stabilize HIF-1α: nuclear HIF-1α was visualized by confocal microscopy; scale bar represents 20 μm. (D) The effect of HIF-1α inhibitor NSC (1 μM) on HepG2 migration and tight junction integrity. Cells were treated with NSC for 24 h and the difference in migration and tight junction integrity determined. (E) HIF-1α staining in normal and HCV infected liver tissue, where the arrow indicates nuclear expression. ^∗∗^*p* <0.01, ^∗∗∗^*p* <0.001.

Altered tight junction protein location is frequently linked with an invasive tumor phenotype and we therefore studied the effect of viral glycoproteins on HepG2 migration and invasion. HepG2 cells expressing HCV glycoproteins demonstrated a significantly increased migratory capacity compared to parental or VSV-G expressing cells (Fig. 3A). Consistent with this increased migratory capacity of HCV glycoprotein containing cells, expression of the cell adhesion molecule, E-Cadherin, was reduced (Fig. 3B). To ascertain whether HCV glycoproteins promote a de-differentiation process reminiscent of EMT, we investigated expression of EMT-associated transcription factors, Snail and Twist. HCV glycoproteins promoted Snail and Twist expression (Fig. 3B). Since these transcription factors are regulated by HIF-1α; a transcription factor associated with the expression of genes involved in tumor invasion, we studied the effect(s) of HCV glycoproteins on HIF-1α expression. Both HCV glycoprotein strains promoted HIF-1α expression under normoxic conditions (Fig. 3C). Treating cells with HIF-1α inhibitor NSC reduced HIF-1α expression (Supplementary Fig. 2) and restored the migratory capacity and tight junction integrity of HCV glycoprotein expressing cells to levels observed with parental and VSV-G expressing cells (Fig. 3D). Treating HepG2 cells with desferrioxamine, an iron chelator that stabilizes HIF-1α, increased hepatoma migration threefold, demonstrating a role for HIF-1α in hepatoma migration independent of HCV infection. Importantly, we observed hepatocellular HIF-1α expression in chronic HCV infected liver tissue (Fig. 3E), demonstrating a focal nuclear staining pattern that was absent in normal liver samples. In summary, HCV glycoproteins increase HepG2 migration and promote the expression of EMT transcription factors Snail and Twist via a HIF-1α driven pathway.

### HCV infection promotes hepatoma migration in a HIF-1α dependent manner

**Supplementary Fig. 3:**
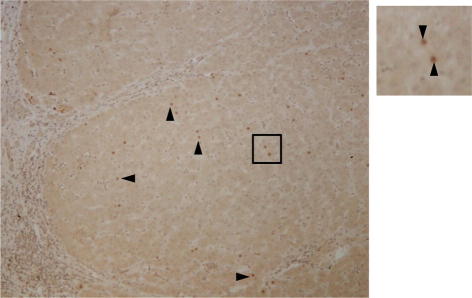
**HIF-1α is stabilized in HCV infected liver tissue.** HIF-1α localization in HCV infected liver tissue. Representative image of HIF-1α distribution in a cirrhotic nodule, arrow heads indicate the nuclear localization of HIF-1α. Box highlights a magnified area of the image.

**Fig. 4:**
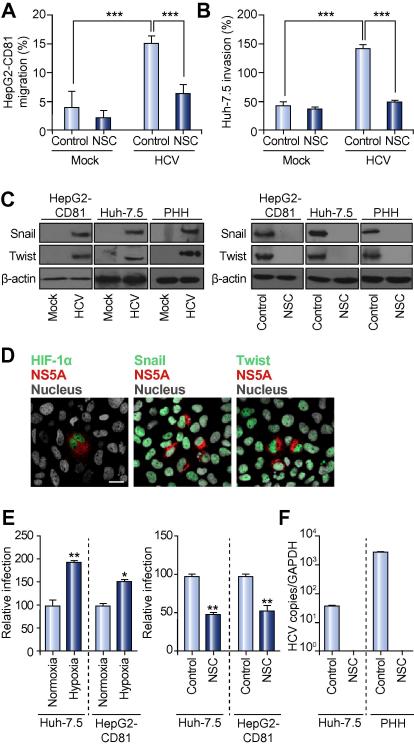
**HCV infection promotes hepatoma migration in a HIF-1α dependent manner.** HepG2-CD81 (A) and Huh-7.5 (B) cells were infected with HCVcc J6/JFH-1 and their migratory capacity after 48 h assessed in a scratch wound assay and collagen invasion assay respectively, in the presence or absence of NSC (1 μM). Data is presented relative to control untreated cells. The number of NS5A expressing HepG2-CD81 and Huh-7.5 cells per well was 1.7 × 10^3^ and 9 × 10^4^, respectively. (C) Snail and Twist expression in mock and HCV infected hepatoma and primary human hepatocytes (PHHs), where NSC treatment ablated Snail and Twist expression. Infected PHHs and Huh-7.5 cells contained 4.1 × 10^5^ HCV RNA copies/10^5^ cells and 3.2 × 10^7^ HCV RNA copies/10^5^ cells, respectively. (D) Huh-7.5 cells were infected with HCV J6/JFH-1 at low multiplicity of infection (0.01) for 48 h and co-stained for NS5A (red), HIF-1α, Snail and Twist (green), and nuclei counterstained with DAPI (gray); scale bar represents 20 μm. (E) The effect of hypoxia (1% O_2_) on HCV infection of hepatoma cells; cells were infected under hypoxic or normoxic conditions and infection determined 48 h later by enumerating NS5A expressing cells, data is presented relative to normoxic conditions. Treating infected cells with HIF-1α inhibitor NSC (1 μM) reduced viral infection; data is presented relative to untreated cells. (F) HCV replication in Huh-7.5 cells and PHHs in the presence or absence of HIF-1α inhibitor NSC (1 μM). Data is presented as HCV copy numbers relative to GAPDH. ^∗^*p* <0.05, ^∗∗^*p* <0.01, ^∗∗∗^*p* <0.001.

To validate these observations with the infectious virus, we assessed the effect(s) of HCVcc strain J6/JFH-1 infection on hepatoma migration using two model systems: a scratch wound assay that measures cell migration within the context of a monolayer and a collagen invasion assay that quantifies hepatoma migration through an extracellular matrix. HCVcc infection significantly increased HepG2-CD81 and Huh-7.5 migration (Fig. 4A and B) and promoted Snail and Twist expression in both hepatoma cell lines and primary human hepatocytes (Fig. 4C). Furthermore, HIF-1α inhibitor NSC restored the migratory capacity of infected hepatoma cells to parental levels and ablated Snail and Twist expression (Fig. 4A–C), confirming a HIF-1α dependent process. We previously reported on the low permissivity of HepG2 cells to support HCV replication [19], raising questions as to how a small number of infected cells affect the migration of the cell population under study. To address this question, the highly permissive Huh-7.5 hepatoma cell line was infected with a low level of infectious virus to generate distinct populations of infected and uninfected cells. HIF-1α was only detected in NS5A antigen expressing cells in the infected culture (Fig. 4D), consistent with the focal HIF-1α staining observed in HCV infected liver tissue (Supplementary Fig. 3). In contrast, the majority of cells in the infected population expressed Snail and Twist, independent of viral antigen expression, suggesting a bystander effect (Fig. 4D).

**Supplementary Fig. 4:**
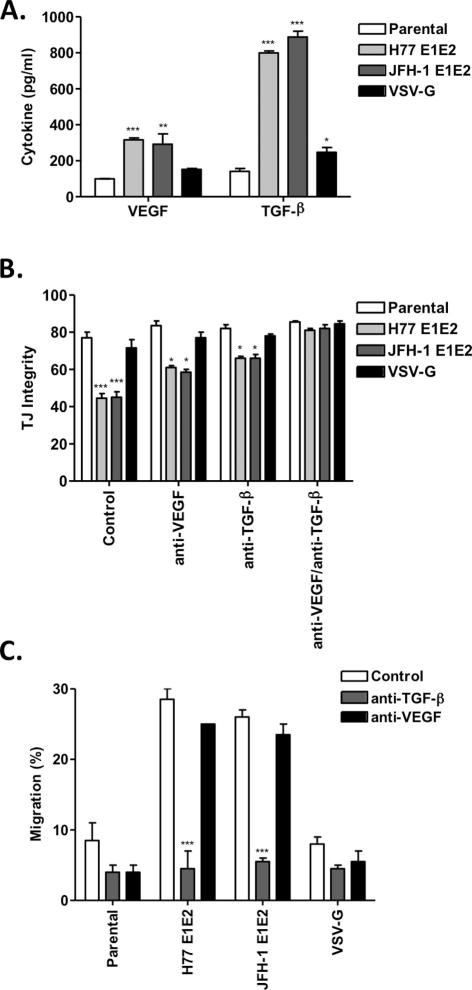
**A role for VEGF and TGF-β in regulating tight junction integrity and hepatoma migration.** (A) VEGF and TGF-β expression per 4x10^4^ cells over a 24 h time period. (B) Polarized cells were treated with anti-VEGF (1.3 μg/ml) or anti-TGF-β (1.5 μg/ml) neutralizing antibodies for 24 h and tight junction integrity (TJ) enumerated. (C) The effect of anti-VEGF (1.3 μg/ml) or anti-TGF-β (1.5 μg/ml) neutralizing antibodies on HepG2 migration. Cells were treated with the respective compounds for 24 h and differences in migration calculated.

**Supplementary Fig. 5:**
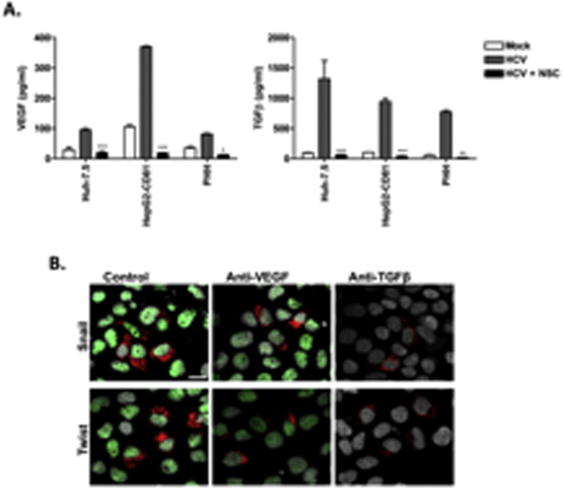
**HIF-1a drives VEGF and TGFb expression in HCV infected hepatoma cells.** (A) NSC ablates enhanced VEGF and TGFβ expression in HCV infected cells. Cells were infected with HCVcc J6/JFH-1 for 48 hours in the presence or absence of NSC (1 μm) and media harvested for cytokine quantification. HCV infected Huh-7.5, primary hepatocytes (PHHs) and HepG2-CD81 cells contained 3.4 x 10^7^, 4.1 x 10^5^ and 3.5 x 10^5^ HCV RNA copies/10^5^ cells, respectively. (B) Anti-TGFβ neutralizes Snail/Twist expression in HCV infected cells. Huh-7.5 cells were infected with J6/JFH-1 at a low multiplicity of infection (0.01) in the presence or absence of neutralising antibodies to VEGF (1.3 μg/ml) or TGFβ (1.5 μg/ml). Cells were co-stained for HCV NS5A (red) and Snail/Twist (green), the nucleus in shown in grey. Scale bar represents 20μm. ∗*p* <0.05, ∗∗ *p* <0.01,∗∗∗ *p* <0.001.

Recent studies have shown that HCV infection stabilizes HIF-1α expression via an ER-stress response that activates PI3K, MAPK and NFKB pathways, leading to increased VEGF and TGFβ expression [22–25]. To ascertain whether HCV glycoproteins promote VEGF and TGFβ expression, parental and glycoprotein expressing cells were analyzed for cytokine expression. There was a significant increase in both VEGF and TGFβ expression in HCV glycoprotein expressing cells (Supplementary Fig. 4A). Neutralizing antibodies targeting VEGF or TGFβ partially restored tight junction integrity in HCV glycoprotein expressing cells, where neutralizing both cytokines resulted in a phenotype indistinguishable from the parental cells (Supplementary Fig. 4B). Importantly, neutralizing TGFβ reduced the migration of HCV glycoprotein expressing cells to levels seen with parental and VSV-G expressing cells (Supplementary Fig. 4C). In contrast, neutralizing VEGF had no detectable effect on hepatoma migration (Supplementary Fig. 4C), consistent with experiments showing that exogenous TGFβ promotes HepG2 migration whereas VEGF had no effect. We confirmed that HCV infection promotes VEGF and TGFβ expression [22–25] and NSC treatment ablated growth factor expression (Supplementary Fig. 5A). Furthermore, neutralizing TGFβ ablated Snail and Twist expression in both NS5A expressing and non-expressing cells in the infected population (Supplementary Fig. 5B), confirming a role for TGFβ in EMT transcription factor expression.

Since low oxygen or hypoxia is known to stabilize HIF-1α, we investigated the effects of hypoxia on HCV replication. Hypoxia significantly increased HCVcc infection of Huh-7.5 and HepG2-CD81 cells and treating cultures with NSC reduced viral infection and had no effect on HCV pseudoparticle entry (Fig. 4E, data not shown). We confirmed these observations with primary human hepatocytes where NSC significantly reduced HCV RNA levels (Fig. 4F), demonstrating a positive role for this transcription factor in the HCV lifecycle. Treating hepatoma cells with an independent HIF-1α inhibitor, RITA, confirmed these findings (data not shown). Taken together, these data provide a new paradigm for HCV to modulate HIF-1α dependent pathways that promote HCC growth and HCV replication.

## Discussion

Our studies show that HCV infection and the viral encoded glycoproteins reduce hepatoma polarity and increase cell migration by stabilizing HIF-1α expression and upregulating downstream effectors, VEGF and TGFβ. Neutralization of both growth factors, or inhibition of HIF-1α, restored the polarity and migratory capacity of infected cells. Furthermore, inhibiting HIF-1α significantly reduced HCV replication in hepatoma cell lines and primary hepatocytes, highlighting a dual role for this transcription factor in the viral life cycle and hepatoma migration.

HIF-1α expression has been reported to associate with EMT, a reversible developmental process where epithelial cells reduce intercellular adhesion and acquire fibroblastoid properties that promote an invasive and metastatic phenotype [12,26]. EMT plays a major role in the invasive and metastatic potential of human cancers [27,28]. EMT transcription factors Snail and Twist are expressed in 40–70% of HCCs and associate with adherens junction disruption and poor prognosis [29,30]. Similarly, ectopic expression of Snail or Twist in hepatoma cell lines enhances their motility and invasiveness [30,31]. Yang and co-authors reported that increased Twist expression was more frequently observed in HCC associated with HCV infection than with other liver diseases [30]. The poor prognosis of HCC is largely due to the invasive nature of the tumor, with frequent intrahepatic and extrahepatic metastases [3]. Therapeutic options for patients with HCC are limited. Curative approaches, including surgical resection and liver transplantation are attempted in approximately 30% of patients, however, cancer recurrence following surgery is in the order of 60–70% within 5 years [32]. This study provides a potential explanation for the clinical observation that HCV associated HCC is frequently more aggressive [7] and highlights a mechanism for HCV to accelerate the malignant process.

Recent reports demonstrate that HCV promotes TGFβ expression and activates p38MAPK, JNK, ERK and NFκB pathways [23,24]. TGFβ promotes EMT and plays a major role in the dissemination of malignant hepatocytes during HCC progression [33,34]. Our data showing a role for TGFβ in the increased migratory capacity of HCV infected hepatoma cells, supports a role for HCV in promoting tumor spread rather than a direct role in the oncogenic process *per se.* We failed to observe any morphological fibroblast features of infected or HCV glycoprotein expressing hepatoma cells, suggesting a partial de-differentiation process *in vitro.* Mazzocca *et al.* recently reported that inhibition of TGFβ receptor I kinase blocked HCC growth, supporting a rationale for therapeutic targeting of TGFβ signaling in HCV associated HCC [35].

Our observation that VEGF and TGFβ perturb tight junction protein localization and function supports our *in vivo* observations demonstrating a widespread reorganization of Occludin in the diseased human liver. Benedicto and colleagues reported that HCV glycoproteins associate with Occludin and alter protein trafficking in Huh-7 cells [36]. In contrast, we failed to immunoprecipitate Occludin and HCV glycoproteins in a variety of cell lines transduced to express HCV E1E2 or in HCV infected cells (data not shown). Furthermore, VEGF or TGFβ had no detectable effect on the expression or localization of Occludin or other tight junction proteins in Huh-7 cells, suggesting that these cells are refractory to these cytokines [10].

HCV infection is one of the leading indications for liver transplantation and the number of patients requiring transplantation for chronic hepatitis C is increasing. HCV infects the newly transplanted liver in all cases, leading to a more rapidly progressive disease and frequent graft loss [37]. Recurrent HCV is recognized as one of the major challenges facing liver transplantation in the next decade [38]. Currently available antiviral treatments are poorly tolerated and have limited efficacy in patients after transplant and new therapies are urgently required. Injury to the liver at the time of transplantation (i.e. ischaemia reperfusion injury, IRI) has been associated with more aggressive recurrent HCV disease [39], however, the factors governing viral replication rate(s) in the newly transplanted liver are poorly understood. Our demonstration that hypoxia, a key event during hepatic IRI, increases virus replication provides a potential explanation for these clinical observations and highlights the potential value of short term anti-oxidant or HIF-1a inhibitor treatment at the time of transplantation to limit HCV replication.

In summary, we have shown a central role for HIF-1α in stimulating VEGF and TGFβ that alters hepatocyte behavior and promotes malignancy in the HCV infected microenvironment. We have also demonstrated a role for HIF-1α in HCV infection. These findings highlight a potential role for HIF-1α inhibitors as therapeutics in patients with both HCC and HCV infection.

## Authors’ contributions

GKW designed experiments, acquired the data and co-wrote the manuscript. CLB, GMR, NFF and MS provided technical assistance. IAR, ZS, RB and SCA provided expert advice. MA, RM and AD provided reagents. SGH and PB provided study supervision. JAM provided study supervision and co-wrote the manuscript. All authors contributed to the final version of the manuscript.

## Financial support

This work was supported by the Medical Research Council, the Wellcome Trust and NIHR Center for Liver Research.

## Conflict of interest

The authors who have taken part in this study declared that they do not have anything to disclose regarding funding or conflict of interest with respect to this manuscript.

## References

[1] El-Serag H.B., Rudolph K.L. (2007). Hepatocellular carcinoma: epidemiology and molecular carcinogenesis. Gastroenterology.

[2] Kensler T.W., Qian G.S., Chen J.G., Groopman J.D. (2003). Translational strategies for cancer prevention in liver. Nat Rev Cancer.

[3] Llovet J.M., Bruix J. (2008). Molecular targeted therapies in hepatocellular carcinoma. Hepatology.

[4] Farazi P.A., DePinho R.A. (2006). Hepatocellular carcinoma pathogenesis: from genes to environment. Nat Rev Cancer.

[5] Liang Y., Shilagard T., Xiao S.Y., Snyder N., Lau D., Cicalese L. (2009). Visualizing hepatitis C virus infections in human liver by two-photon microscopy. Gastroenterology.

[6] McGivern D.R., Lemon S.M. (2011). Virus-specific mechanisms of carcinogenesis in hepatitis C virus associated liver cancer. Oncogene.

[7] Huang Y.H., Wu J.C., Chen C.H., Chang T.T., Lee P.C., Chau G.Y. (2005). Comparison of recurrence after hepatic resection in patients with hepatitis B vs. hepatitis C-related small hepatocellular carcinoma in hepatitis B virus endemic area. Liver Int.

[8] Wakita T., Pietschmann T., Kato T., Date T., Miyamoto M., Zhao Z. (2005). Production of infectious hepatitis C virus in tissue culture from a cloned viral genome. Nat Med.

[9] Burlone M.E., Budkowska A. (2009). Hepatitis C virus cell entry: role of lipoproteins and cellular receptors. J Gen Virol.

[10] Mee C.J., Farquhar M.J., Harris H.J., Hu K., Ramma W., Ahmed A. (2010). Hepatitis C virus infection reduces hepatocellular polarity in a vascular endothelial growth factor-dependent manner. Gastroenterology.

[11] Orban E., Szabo E., Lotz G., Kupcsulik P., Paska C., Schaff Z. (2008). Different expression of Occludin and ZO-1 in primary and metastatic liver tumors. Pathol Oncol Res.

[12] van Zijl F., Zulehner G., Petz M., Schneller D., Kornauth C., Hau M. (2009). Epithelial–mesenchymal transition in hepatocellular carcinoma. Future Oncol.

[13] Turksen K., Troy T.C. (2011). Junctions gone bad: claudins and loss of the barrier in cancer. Biochim Biophys Acta.

[14] Zhang J., Randall G., Higginbottom A., Monk P., Rice C.M., McKeating J.A. (2004). CD81 is required for hepatitis C virus glycoprotein-mediated viral infection. J Virol.

[15] Mitry R.R. (2009). Isolation of human hepatocytes. Methods Mol Biol.

[16] Hsu M., Zhang J., Flint M., Logvinoff C., Cheng-Mayer C., Rice C.M. (2003). Hepatitis C virus glycoproteins mediate pH-dependent cell entry of pseudotyped retroviral particles. Proc Natl Acad Sci U S A.

[17] Chau N.M., Rogers P., Aherne W., Carroll V., Collins I., McDonald E. (2005). Identification of novel small molecule inhibitors of hypoxia-inducible factor-1 that differentially block hypoxia-inducible factor-1 activity and hypoxia-inducible factor-1alpha induction in response to hypoxic stress and growth factors. Cancer Res.

[18] Yang J., Ahmed A., Poon E., Perusinghe N., de Haven Brandon A., Box G. (2009). Small-molecule activation of p53 blocks hypoxia-inducible factor 1alpha and vascular endothelial growth factor expression in vivo and leads to tumor cell apoptosis in normoxia and hypoxia. Mol Cell Biol.

[19] Mee C.J., Harris H.J., Farquhar M.J., Wilson G., Reynolds G., Davis C. (2009). Polarization restricts hepatitis C virus entry into HepG2 hepatoma cells. J Virol.

[20] Reynolds G.M., Harris H.J., Jennings A., Hu K., Grove J., Lalor P.F. (2008). Hepatitis C virus receptor expression in normal and diseased liver tissue. Hepatology.

[21] Lindenbach B.D., Evans M.J., Syder A.J., Wolk B., Tellinghuisen T.L., Liu C.C. (2005). Complete replication of hepatitis C virus in cell culture. Science.

[22] Nasimuzzaman M., Waris G., Mikolon D., Stupack D.G., Siddiqui A. (2007). Hepatitis C virus stabilizes hypoxia-inducible factor 1alpha and stimulates the synthesis of vascular endothelial growth factor. J Virol.

[23] Presser L.D., Haskett A., Waris G. (2011). Hepatitis C virus-induced furin and thrombospondin-1 activate TGF-beta1: role of TGF-beta1 in HCV replication. Virology.

[24] Lin W., Tsai W.L., Shao R.X., Wu G., Peng L.F., Barlow L.L. (2010). Hepatitis C virus regulates transforming growth factor beta1 production through the generation of reactive oxygen species in a nuclear factor kappaB-dependent manner. Gastroenterology.

[25] Hassan M., Selimovic D., Ghozlan H., Abdel-kader O. (2009). Hepatitis C virus core protein triggers hepatic angiogenesis by a mechanism including multiple pathways. Hepatology.

[26] Thiery J.P., Acloque H., Huang R.Y., Nieto M.A. (2009). Epithelial–mesenchymal transitions in development and disease. Cell.

[27] Hanahan D., Weinberg R.A. (2011). Hallmarks of cancer: the next generation. Cell.

[28] Choi S.S., Diehl A.M. (2009). Epithelial-to-mesenchymal transitions in the liver. Hepatology.

[29] Sun T., Zhao N., Zhao X.L., Gu Q., Zhang S.W., Che N. (2010). Expression and functional significance of Twist1 in hepatocellular carcinoma: its role in vasculogenic mimicry. Hepatology.

[30] Yang M.H., Chen C.L., Chau G.Y., Chiou S.H., Su C.W., Chou T.Y. (2009). Comprehensive analysis of the independent effect of twist and snail in promoting metastasis of hepatocellular carcinoma. Hepatology.

[31] Matsuo N., Shiraha H., Fujikawa T., Takaoka N., Ueda N., Tanaka S. (2009). Twist expression promotes migration and invasion in hepatocellular carcinoma. BMC Cancer.

[32] Llovet J.M. (2005). Updated treatment approach to hepatocellular carcinoma. J Gastroenterol.

[33] Giannelli G., Bergamini C., Fransvea E., Sgarra C., Antonaci S. (2005). Laminin-5 with transforming growth factor-beta1 induces epithelial to mesenchymal transition in hepatocellular carcinoma. Gastroenterology.

[34] Fransvea E., Angelotti U., Antonaci S., Giannelli G. (2008). Blocking transforming growth factor-beta up-regulates E-Cadherin and reduces migration and invasion of hepatocellular carcinoma cells. Hepatology.

[35] Mazzocca A., Fransvea E., Lavezzari G., Antonaci S., Giannelli G. (2009). Inhibition of transforming growth factor beta receptor I kinase blocks hepatocellular carcinoma growth through neo-angiogenesis regulation. Hepatology.

[36] Benedicto I., Molina-Jimenez F., Barreiro O., Madonado-Rodriguez A., Prieto J., Moreno-Otero R. (2008). Hepatitis C virus envelope components alter localization of hepatocyte tight junction-associated proteins and promote Occludin retention in the endoplasmic reticulum. Hepatology.

[37] Ramirez S., Perez-Del-Pulgar S., Carrion J.A., Costa J., Gonzalez P., Massaguer A. (2009). Hepatitis C virus compartmentalization and infection recurrence after liver transplantation. Am J Transplant.

[38] Rowe I.A., Barber K.M., Birch R., Curnow E., Neuberger J.M. (2010). Retransplantation for graft failure in chronic hepatitis C infection: a good use of a scarce resource?. World J Gastroenterol.

[39] McCaughan G.W., Shackel N.A., Bertolino P., Bowen D.G. (2009). Molecular and cellular aspects of hepatitis C virus reinfection after liver transplantation: how the early phase impacts on outcomes. Transplantation.

